# Adipokines as biochemical marker of polycystic ovary syndrome in adolescents – review

**DOI:** 10.3389/fendo.2025.1475465

**Published:** 2025-05-26

**Authors:** Dominika Orszulak, Kacper Niziński, Aleksandra Matonóg, Maja Zięba-Domalik, Rafał Stojko, Agnieszka Drosdzol-Cop

**Affiliations:** Department of Gynecology, Obstetrics and Oncological Gynecology, Faculty of Health Sciences in Katowice, Medical University of Silesia, Katowice, Poland

**Keywords:** polycystic ovary syndrome, adipokines, adolescents, leptin, adiponectin

## Abstract

Polycystic ovary syndrome (PCOS) is one of the most common endocrine disorders affecting approximately 10% of adolescent women. According to literature data, in the case of coexistence of obesity and PCOS, menstrual cycle disorders are much more common. One of the probable reasons is the activity of adipose tissue, which secretes many types of adipokines that may have a negative impact on hormonal and metabolic functions. This article reviews the literature on the role of adipokines in the etiopathogenesis of PCOS in adolescent girls. The literature base consisted of published articles - clinical trials, meta-analyses, reviews and systematic reviews. The databases - PubMed, ScienceDirect, EMBASE were searched.

## Introduction

Adipose tissue (AT) synthesizes and secretes many biologically active proteins adipokines with autocrine, paracrine, and endocrine properties. AT is traditionally classified into white adipose tissue (WAT), and brown adipose tissue (BAT), with WAT constituting approximately 95% of total AT mass. WAT itself is further divided into subcutaneous adipose tissue (SAT) and visceral adipose tissue (VAT), which differ in anatomical location, metabolic activity, and secretory profile. SAT, located beneath the skin, is the predominant source of leptin and adiponectin, which are adipokines with anti-inflammatory properties and insulin-sensitizing effects. In contrast, VAT, situated around internal organs, displays higher lipolytic activity and greater secretion of pro-inflammatory adipokines such as tumor necrosis factor-alpha (TNF-α) and interleukin-6 (IL-6), contributing to insulin resistance and an increased risk of cardiometabolic diseases. BAT, which constitutes about 2% of total AT, is specialized in thermogenesis and secretes batokines that enhance energy expenditure and improve insulin sensitivity. The distinct endocrine profiles of these adipose depots underscore their differential contribution to systemic metabolic regulation and are of particular relevance to the pathophysiology of polycystic ovary syndrome (1, 2).

Overweight and obesity among adolescents is an epidemically growing problem, the effects of which are reflected in adulthood. Data show that over the past 30 years, there has been a twofold increase in the prevalence of obesity among children and a fourfold increase among adolescents. During adolescence, obesity may be an early indication of hypothalamic-pituitary-ovarian axis dysfunction and ultimately lead to abnormal ovarian function in reproductive age, in the form of menstrual disorders (oligomenorrhea, amenorrhea, heavy menstrual bleeding), PCOS, infertility and endometrial abnormalities - endometrial polyps, proliferation with/without atypia of the endometrium and endometrial carcinoma (3, 4).

PCOS is one of the most common endocrinopathies, affecting approximately 10% of the girl population. The heterogeneity of symptoms, endocrine and metabolic abnormalities in this disease entity, makes it very difficult to establish the aetiopathogenesis of PCOS (5).

There is a complex relationship between PCOS and obesity. Obesity in PCOS, not only results in an exacerbation in the clinical symptoms of the syndrome, but also leads to metabolic disorders. The literature reports that in the population of women with obesity, the prevalence of PCOS increases to approximately 30% (6–8). Wood et al. in their review of the literature, confirm the connection between obesity and PCOS - up to 84% of PCOS patients are thought to be obese (2). Christensen et al. conducted a study involving 137,502 adolescent girls aged 15–19 years. The results show that the prevalence of PCOS according to the National Institutes of Health (NIH) criteria was approximately 3.0, 6.7, and 14.7 times higher in girls with overweight, moderate obesity, and extreme obesity, respectively. Furthermore, it was observed that adolescent girls with PCOS were more likely to be obese than their healthy peers (63.1% vs. 16.5%) (9).

Girls with obesity and polycystic ovary syndrome are significantly more likely to have heavy menstrual bleeding and iron deficiency anemia as a result of higher blood serum estrogen concentrations and endometrial hyperplasia, as well as increased clinical signs of hyperandrogenism, higher total and free testosterone levels, lower sex hormone binding globulin (SHBG) levels and compensatory hyperinsulinemia with insulin resistance (2).

In the literature, we find data that patients with obesity and PCOS are more likely to have menstrual cycle disorders compared to normal weight patients. Additionally, in PCOS, central obesity is usually observed, which is associated with a higher risk of insulin resistance and hyperandrogenemia (2, 7, 10). Furthermore, obesity is an independent risk factor for endocrine disruption: elevated blood serum total testosterone and insulin levels and reduced SHBG concentration levels (2, 7, 10).

In the past, adipose tissue was perceived solely as an energy reservoir, but numerous studies have shown that it is a source of numerous substances that affect metabolic processes, constituting an axial disorder in type 2 diabetes, metabolic syndrome or PCOS (1). Abnormal adipokines secretion as a result of inflammation within the adipose tissue may significantly contribute to endocrine and metabolic abnormalities in PCOS (11, 12). Adipokines play an important role in the aetiopathogenesis of PCOS and their exact part remains unclear and requires further research.

The aim of this review is to determine the role of adipokines in the aetiopathogenesis and clinical picture of PCOS in adolescent girls. English-language publications were included by searching the database - PubMed, ScienceDirect, EMBASE, using the terms - adolescent girls, PCOS, adipokines, leptin, adiponectin, resistin, apelin, vaspin, visfatin, ghrelin, omentin, obesity, insulin resistance, metabolic syndrome.

## Leptin

Leptin is the best-studied adipokine, discovered in 1994 by cloning the obesity gene in mice. It is a 16 kDa protein hormone, encoded by the OB gene on chromosome 7, and is mainly produced by differentiated adipocytes. Leptin receptors are located in the central nervous system - the hypothalamus, the choroid plexus of the brain and in peripheral tissues such as the ovary, placenta, adrenal glands and thyroid gland (13, 14).

Leptin concentration changes according to circadian rhythm, reaching a maximum between 10 p.m. and 3 a.m., which is related to its effect on the satiety and hunger center in the hypothalamus. It inhibits appetite by reducing the synthesis of neuropeptide Y. Due to its properties, it is often referred to as an anorexigenic hormone or satiety hormone. Moreover, its concentration increases with fat mass and changes during the menstrual cycle - with a peak during the luteal phase (15, 16).

Leptin directly affects gonadoliberin (GnRH) release, gonadotropin secretion (increases follitropin (FSH), lutropin (LH), thyrotropin (TSH) production and release, inhibits somatotropin (GH) and adrenocorticotropic hormone (ACTH) production and ovarian steroidogenesis. Literature reports support the relation of hypoleptinemia with female infertility and delayed puberty. In addition, leptin is involved in other reproductive functions: lactation, folliculogenesis, oocyte growth and maturation. In hepatocytes, it enhances the inhibitory effect of insulin on hepatic gluconeogenesis, and in pancreatic beta cells, it inhibits insulin secretion (17).

In a study by Kale-Gurbuza et al. (2013) 38 adolescent girls with obesity, 17 of whom were diagnosed with PCOS were examined. Leptin and ghrelin levels were similar in the group of girls with obesity and PCOS and the control group with obesity alone. According to this study, leptin positively correlates with body mass index (BMI), oestradiol and TSH levels. Also, a metanalysis by Li et al. (2017) showed that, compared to normal-weight adolescents with PCOS, a group of adolescents with obesity and PCOS had significantly lower levels of SHBG and HDL-C and significantly higher levels of triglycerides, leptin, fasting insulin, LDL-C and free testosterone (13, 18).

Interestingly, an article by Y Al-Taee et al. (2022) showed that elevated leptin and prolactin levels in women with PCOS modify immune mechanisms in these women (19).

Zukauskaite et al. in their study showed higher levels of leptin in adolescents with hyperandrogenism compared to the control group, which may indicate the potential involvement of this adipokine in the development of PCOS (20).

## Adiponectin

Adiponectin is a polypeptide hormone secreted by adipose tissue. Its synthesis is stimulated by: insulin, PPAR-γ receptor agonists, and inhibited by: TNF-α and PPAR-α receptor agonists (21).

In obesity, there is a reduced production of adiponectin (which enhances the proinflammatory effects of TNF-α), while its concentration increases with decreasing body weight. We find reports in the literature that adiponectin concentrations may decrease during puberty (22).

Adiponectin has antidiabetic properties - it increases insulin sensitivity by inhibiting hepatic gluconeogenesis and increasing glucose consumption in muscles (21, 23). In addition, as a result of increased fatty acid oxidation, it exerts an antiatherosclerotic effect and has a beneficial effect on lipid metabolism - lowering fatty acid and triglyceride concentrations in the blood. Many studies suggest that adiponectin levels are positively correlated with HDL fraction cholesterol levels, and negatively correlated with fasting blood glucose, blood pressure, insulinemia, LDL fraction cholesterol and triglyceride levels (24–26).

A higher concentration of adiponectin is observed in women than in men. Estradiol positively correlates with adiponectin levels and stimulates the expression of its receptors, whereas testosterone has the opposite effect. The results of studies conducted by Spranger et al. showed a negative correlation between adiponectin and testosterone in patients with PCOS (27). Moreover, Comim et al. demonstrated that a reduction in the expression of adiponectin receptors—AdipoR1 and AdipoR2—leads to an increased synthesis of androstenedione by ovarian theca cells, suggesting the involvement of adiponectin in the etiopathogenesis of hyperandrogenism in PCOS (28).

Riestra et al. demonstrate that adiponectin levels in adolescent girls are positively correlated with SHBG levels and negatively correlated with free androgen index. The authors suggest that this association is independent of BMI values and body fat mass (29). Adiponectin has also been shown to inhibit the release of LH and GH, without affecting FSH levels, and has an effect on ovarian steroidogenesis (30). Furthermore, adiponectin is negatively correlated with the insulin resistance index in adolescent girls with PCOS (31).

Moreover, adiponectin concentrations are lower in a group of adolescent girls with obesity and PCOS compared to a group of normal-weight girls with PCOS (32). Yasar et al. suggest that the concentration of the studied adipokine is significantly lower among adolescent girls with PCOS compared to controls, but is independent of BMI (33). Data in the literature are divergent, and the exact dependence of adiponectin levels in PCOS in adolescent girls requires further study (32, 33).

Cekmez et al. in their study, observed significantly reduced adiponectin levels in a group of girls with obesity PCOS and elevated values of the insulin resistance index, HOMA-IR, compared to girls without PCOS (12). Toulis and Yilmaz observed hypoadiponectinemia among mature women with PCOS, both in the normal weight and abnormal BMI groups, and reduced levels of the studied adipokine are likely to be associated with insulin resistance among patients with PCOS. Many researchers suggest that hypoadiponectinemia is an important factor linking obesity, insulin resistance and PCOS (34, 35). The results of several studies support the hypothesis that impaired adiponectin secretion by adipose tissue, combined with insulin resistance, plays an important role in the early development of PCOS among adolescent girls with obesity, and that hypoadiponectinemia may be a new biomarker of insulin sensitivity (12).

A different view is presented by Pinkas-Himel, claiming that reduced adiponectin concentrations only in a group of girls with obesity and PCOS, excludes the involvement of this adipokine in the aetiopathogenesis of PCOS, and that its concentration inversely correlates with BMI and leptin and insulin concentrations (32). A similar view is presented by Kale-Gurbuz, who draws the conclusion that PCOS is not a factor influencing adiponectin concentrations in girls with obesity (13).

Güven on the basis of her study believes that hypoadiponectinemia in girls with obesity and PCOS may be a risk factor for cardiovascular diseases - dyslipidemia, hypertension, insulin resistance (36).

We find few data in the literature identifying an association between adiponectin and PCOS in normal-weight women/teenagers. A case-control study by Mirza et al. (2014) involving normal-weight women (BMI<23) aged 16–35 years with PCOS showed that adiponectin may be an independent diagnostic marker for PCOS in young normal-weight patients with less severe clinical symptoms of the syndrome or in a group of women with a positive family history of PCOS, as its levels are independently associated with PCOS (37).

## Omentin

Omentin, another of the adipokines, was discovered in 2005 and exists in the form of two isoforms, omentin-1 (interlectin-1) and omentin-2. The omentin gene is located on chromosome 1q22-q23, being expressed primarily in visceral adipose tissue stromal cells, as well as in intestinal Paneth cells, endothelial cells, macrophages, fibroblasts and epicardium (38).

Omentin exhibits anti-inflammatory effects and sensitises tissues to insulin action, and its reduced levels are a risk factor for metabolic diseases. According to the literature, insulin resistance is the primary metabolic disorder in women with PCOS, independent of the patients’ BMI (39, 40).

Data in the literature on the relationship of omentin with PCOS in adolescent girls are very limited. We find only one publication in the literature for this age group. A study involving 41 girls with obesity and PCOS showed lower omentin-1 levels in adolescent girls with obesity and PCOS compared to girls with obesity but without PCOS, as well as a negative correlation with free testosterone levels. However, the relationship between omentin-1 and the HOMA-IR index was not proven (41). The results obtained are consistent with data in the literature - Choi et al. also observed reduced omentin-1 levels in women with PCOS, regardless of BMI (42). However, a number of studies show a negative correlation of the investigated adipokine with HOMA-IR, indicating the need for further research in this age group (40, 43, 44). Previous findings suggest that a variable adipokine profile, including a decrease in omentin-1 levels in PCOS patients, may be a consequence of hyperandrogenism (41).

## Vaspin

Vaspin (VASP) is a relatively new adipokine discovered in 2005. It is encoded by the SERPINA12 gene, located on the long arm of chromosome 14, and is associated with the development of insulin resistance, obesity and inflammation. Interestingly, its expression has been found not only in visceral and subcutaneous adipose tissue, but also in the skin, liver, pancreas, placenta, stomach, cerebrospinal fluid, hypothalamus and ovaries (45).

VASP is assumed to have a hypoglycemic effect and to increase tissue insulin sensitivity, although the molecular mechanisms of its action are not well understood (45). Based on a study of 89 children, Gajewska et al. suggest that polymorphisms of the rs2236242 gene encoding vaspin affect body composition and lipid profile in prepubertal children, which may affect the risk of obesity and related diseases in the future (46). In addition to functions characteristic for other adipokines, such as regulation of proliferation, apoptosis or angiogenesis processes, it also influences preadipocyte differentiation, steroid synthesis, oocyte maturation and corpus luteum formation (45).

Despite the abundance of data, we find few papers in the literature on VASP expression in adipose tissue and blood serum concentrations of this adipokine in children with obesity and in a group of girls with PCOS. The results of studies in this age group are very divergent.

Cekmez et al. analyzing VASP concentrations in a group of 48 obese, adolescent girls with PCOS found that the average vaspin concentration in this group was higher than in the adolescents in the control group, but the difference was not statistically significant. All other studies referred overwhelmingly to girls with obesity and overweight, fatty liver and carbohydrate metabolism disorders (12).

Özkan et al. published a paper in 2022 investigating adipokine levels and their correlation with the degree of hepatic steatosis in children with overweight and overweight. 81 children with a BMI above the 95th percentile were recruited into the study group, while 32 normal-weight children were recruited into the control group. In conclusion, it was found that VASP levels in children with hepatic steatosis and elevated BMI were lower than in children in the control group. Furthermore, a positive correlation between HDL and vaspin levels was noted. However, there was no significant change in VASP levels depending on the degree of hepatic steatosis in the affected children (47). Reinherr and Roth, on the other hand, include vaspin as a pro-inflammatory cytokine that is associated with insulin resistance and metabolic syndrome (MS) in children, which contradicts the results of the aforementioned study (48). Buyukinan et al. in a study involving 47 children with obesity and metabolic syndrome (MS) also observed higher levels of VASP in the study group and its strong correlation with high CRP values (49). Similar conclusions were also reached by Salama et al. (50). This may support the view that this adipokine in children with metabolic syndrome (MS) contributes to increased inflammatory markers and may precede the onset of abnormal glucose metabolism. Also, Zlatkina, in her 2016 paper, concludes that increased blood serum VASP levels may precede changes in insulin levels in children, and that monitoring of VASP levels may allow a group at high risk of developing metabolic disease and hypertension in the future to be more quickly identified (51). These observations are corroborated by a large Korean study of 168 overweight boys and 176 overweight girls. It proved that VASP concentrations in the overweight group of children were significantly higher than in the normal-weight group, while showing a positive correlation with body weight, BMI and diastolic blood pressure (52). Suleymanoglu et al. additionally showed the same correlation with BMI, triglyceride levels, insulin and HOMA-IR. However, a negative correlation with adiponectin and fasting glucose/insulin ratio was reported (53).

The studies by Körner et al. and Martos-Moreno do not fully confirm the above results. Körner et al. marked blood serum VASP levels in 67 children with obesity. The control group included 65 normal-weight children. In this study, there was no correlation between blood VASP concentrations and BMI found. However, significantly higher blood serum vaspin concentrations were found in girls compared to boys. Girls with obesity had significantly lower blood VASP concentrations compared to normal-weight girls, which is in considerable contradiction with the analysis of the previously cited studies. It is also worth mentioning that an increase in VASP concentrations with age and stage of sexual maturation was observed in girls (54, 55).

Similar findings were reached by Martos-Moreno et al. who assessed blood serum VASP concentrations in 100 prepubertal children. They also observed no differences in blood vaspin concentrations between the group of children with obesity and normal-weight. Interestingly, even weight reduction measured by BMI and body fat reduction estimated by DEXA did not result in changes in blood VASP concentrations in these children (55). These results are in contrast to another study on a group of 50 children with overweight and obesity, in whom already a short-term lifestyle modification resulted in a significant decrease in blood serum VASP concentration and an improvement in insulin sensitivity expressed by a decrease in HOMA- IR (56).

The number of publications and scientific studies on vaspin and its effects on the metabolism of children and, in particular, the metabolism of girls during puberty is severely limited. The conclusions drawn from the literature analysis are also ambiguous, as the results of individual studies often contradict each other. Nevertheless, the effects of VASP on carbohydrate metabolism, insulin sensitivity or oocyte maturation seem to be indisputable, and all the metabolic abnormalities mentioned are closely related to PCOS and underlie this disease.

## Resistin

Resistin was first detected in rodents and was initially known as a hormone secreted by adipocytes, which is associated with obesity and insulin resistance. Recent data indicate that in humans, it is mainly produced and secreted by peripheral blood mononuclear cells (PBMC), being a mediator of numerous inflammatory processes. Resistin increases the expression of proinflammatory cytokines such as TNF-α, IL-6, IL-12 and monocyte chemotactic protein (MCP1) and acts by interacting with Toll-like receptor 4 (TLR4) in human myeloid and epithelial cells. This relationship is highly relevant as it links resistin to inflammation and possibly to insulin resistance, making it of interest in studies related to metabolic disorders such as diabetes and obesity. Currently, both obesity and type 2 diabetes mellitus (T2DM) and cardiovascular diseases (CVD) have been recognized as chronic inflammatory diseases that are most likely linked to the action of cytokines and adipokines, including resistin (57–59).

Numerous studies indicate that increased resistin levels are positively correlated with the development of insulin resistance, diabetes and cardiovascular diseases such as dyslipidemia, atherosclerosis and hypertension. Its elevated concentrations are also observed in non-alcoholic fatty liver disease (NAFLD) and inflammatory conditions of this organ (57–59).

The role of resistin in the pathophysiology of PCOS is widely debated, but the literature does not provide conclusive evidence for its significant role in the development of this endocrinopathy. Some authors have studied blood serum resistin levels in patients with PCOS, but the results of these studies so far remain contradictory (59). In 2021, Raeisi et al. in order to clarify the discrepancies regarding resistin and follistatin levels in women with PCOS conducted a meta-analysis of the scientific literature on the subject matter. Thirty-eight publications covering a total of 2424 cases of patients with PCOS and 1906 patients in control groups were included in the study. It was unequivocally found that resistin levels were significantly higher in patients with PCOS compared to the control group, irrespective of BMI, suggesting that this adipokine may play an important role in the pathogenesis of PCOS (60). Subsequent papers published after this meta-analysis seem to mostly confirm the above conclusions (61, 62).

Data on the role of resistin in the context of PCOS development in adolescent girls are limited. Sartori et al. in 2016 did not find a single paper investigating the effect of resistin on sexual development during adolescence.

In the scientific literature, we find 2 publications linking PCOS in adolescent girls to the adipokine in question. Güven et al. published a study in 2010 that included 22 adolescent girls with PCOS and 16 adolescents without PCOS as controls. The aim of their study was to assess fasting and glucose-loaded blood serum adiponectin and resistin levels and their impact on CVD risk in adolescents with PCOS. Numerous indices of insulin resistance were also calculated, such as HOMA-IR, insulin sensitivity index (QUICKI), fasting glucose-to-insulin ratio (FGIR) and whole-body insulin sensitivity index (ISI). Unfortunately, no correlation between resistin levels and indices of insulin resistance was found in the conducted study (36).

Two years earlier, Bideci et al. also sought to assess resistin levels in adolescent female patients with PCOS and determine its potential correlations with hormonal and metabolic features of the condition. The study included 16 girls with obesity and 12 normal-weight girls with PCOS and 19 adolescent girls with obesity as controls. In the context of resistin, it was only found that its concentration significantly correlates with free testosterone and sex hormone-binding globulin levels in the blood (63).

## Apelin

Apelin is a cytokine, encoded by the APLN gene of the long arm of the X chromosome. The presence of apelin mRNA, as well as apelin receptor mRNA, has been found in: vascular endothelium, central nervous system, gastrointestinal tract and peripheral blood cells (64). There are several forms of apelin: apelin-12, apelin-13, apelin-16, apelin-17, and apelin-36 (65).

Apelin is one of the most potent substances with a positive inotropic effect, exhibiting vasodilatory properties by stimulating nitric oxide synthesis and inhibiting vasopressin secretion (66–68). In the literature, we find data that apelin has an effect on carbohydrate metabolism - it exhibits insulin-like effects by lowering glucose concentrations due to its increased consumption in muscle and adipose tissue (69). Recently, it has been more often suggested that apelin may increase insulin sensitivity and improve insulin resistance (70). Based on their findings, Sorhede et al. suggest that apelin inhibits insulin secretion (71).

In addition, the concentration of this adipokine is dependent on the degree of adipose tissue development. A Polish study showed that apelin concentrations were significantly lower in a group of women with Anorexia nervosa compared to a control group with normal BMI and a group of women with obesity (72). Moreover, some studies show a positive correlation between tumor necrosis factor (TNF- α) levels and apelin together with its positive effect on angiogenesis within adipose tissue (73, 74). A relatively new study in gestational diabetic mice shows that apelin can regulate lipid metabolism by increasing the activity of the PI3K/Akt signaling pathway and play a role in lowering total cholesterol and increasing HDL-C levels (75).

There are few studies in the literature that have assessed the association between apelin levels and endocrine profile and metabolic abnormalities in PCOS among adolescent girls, and the results are inconsistent. Choi et al. observed from their study that apelin levels were significantly lower in PCOS patients compared to controls, and its levels were negatively correlated with free androgen index (FAI) and total testosterone levels (76). Similar conclusions were also reached by Chang et al. and Altinkaya et al., whose studies also showed reduced apelin levels in women with PCOS compared to the control group (77, 78).

Other results were obtained by Olszanecka-Glinianowicz et al. where the concentrations of apelin-12 and apelin-36 were not statistically significantly different in the group of women with PCOS and women without PCOS. However, in a subgroup analysis, they observed reduced apelin-36 levels in patients with obesity, PCOS and elevated levels in the normal-weight group, compared with the control group (79). The authors suggest that the elevated apelin-36 levels in the normal-weight group of women result from mechanisms compensating for the increasing disturbances of carbohydrate metabolism.

In the most recent study from 2023, which recruited 44 adolescents with PCOS and 44 adolescents without PCOS, the authors also observed no difference in apelin concentration between the control and study groups or its association with insulin resistance (80). Analogous conclusions were drawn by Benk et al., who similarly observed no difference in apelin levels between the study groups (81).

A study by Cekmez et al. showed a different relationship - significantly higher apelin values in a group of adolescent girls with PCOS and a positive correlation with BMI and HOMA-IR (12). Their observations were in line with those of Hosoy et al. who, on the basis of their study, noted that apelin levels were clearly increased in patients with insulin resistance and hyperinsulinemia. Furthermore, the group of PCOS patients in their study had higher apelin concentration than the control group, and it positively correlated with HOMA-IR values (82). In addition, at least two other studies have shown significantly elevated apelin levels in PCOS patients (83, 84).

The structure and function of apelin seem to be quite well understood. However, according to an analysis of the scientific literature, information on its role in adolescent girls with PCOS is scarce and the results of studies are divergent. This may be due to the relatively small size of the patient groups recruited for the studies. There is certainly a need for further exploration of the presented topic.

## Visfatin

Another of the adipokines important in the pathogenesis of PCOS is visfatin. It is a protein cytokine consisting of 491 amino acids. It was discovered as a protein involved in pancreatic beta cell maturation and is identical to pre-B-cell colony-enhancing factor (PBEF). Visfatin influences glucose metabolism by affecting pancreatic islet beta cells, thereby modifying oxidative stress-related processes, and is secreted in adipose tissue as well as muscles, liver, kidneys, bone marrow, heart, trophoblast and fetal membranes (59, 80).

Interestingly, current scientific reports show that visfatin (as well as apelin) in PCOS not only plays a hypoglycemic role, but may also exacerbate inflammation, thus worsening insulin resistance. The study by Ruan et al. examined 88 girls aged between 12 and 20 years, with half of the study group burdened with PCOS. They found that visfatin and apelin levels did not differ between the study and control groups. Importantly, however, the researchers found that visfatin is a predictor of insulin resistance, leading to an improved lipid profile and thus may have a protective function in the development of metabolic syndrome in adolescent female patients with PCOS. The results of studies on visfatin levels in PCOS are conflicting, with some finding significantly higher levels of visfatin in the study group compared to controls, while others show, as in the study by Ruan et al. no significant differences between the groups (80).

## Discussion

**Figure 1:**
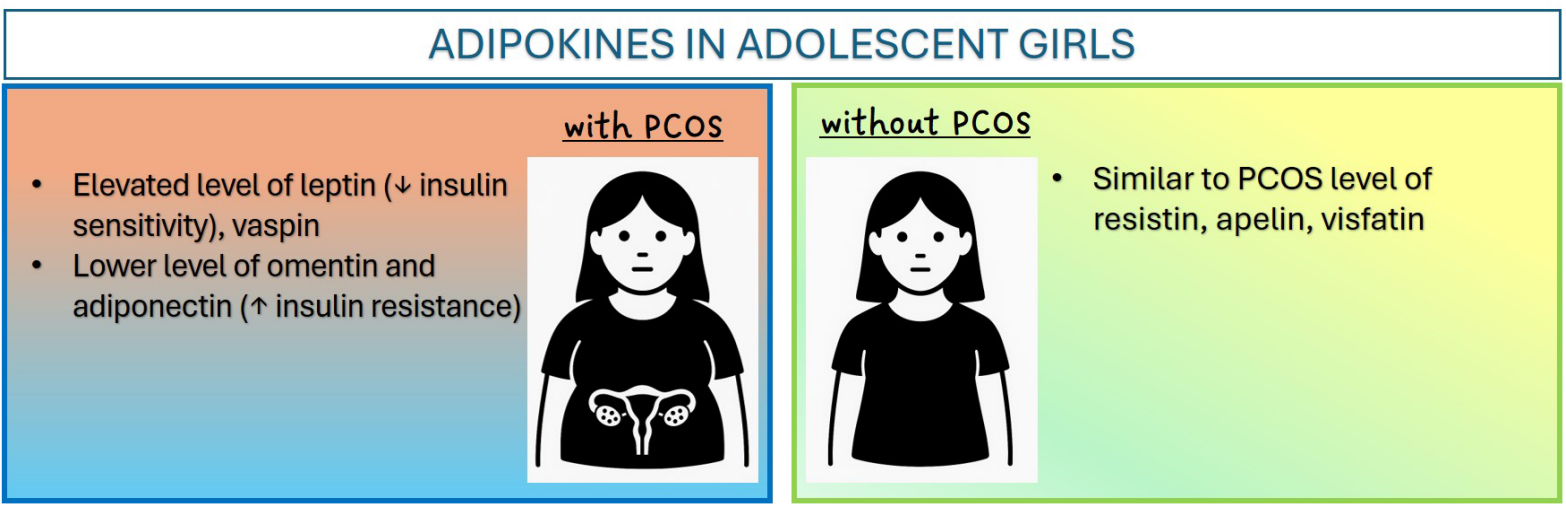
Adipokines in adolescent girls.

The above article demonstrates the influence of adipokines on the etiopathogenesis and clinical picture of PCOS in a group of adolescent girls (**Figure 1**). Environmental, genetic, and epigenetic factors can influence adipose tissue dysfunction; however, regardless of the primary etiological factor, the resulting inflammation within adipose tissue and the reduced insulin sensitivity of adipocytes lead to disturbances in adipokine secretion, creating a vicious metabolic cycle. Most studies focusing on the role of adipose tissue in the development of PCOS have assessed its function indirectly, concentrating solely on measurements of individual adipokine concentrations in the blood. However, it is important to note that many of these bioactive molecules are secreted not only by adipose tissue, which can significantly influence their concentration in the blood. Furthermore, the results of some studies show that the mere presence of PCOS has an impact on adipokine and cytokine levels. Often, in this group of patients, adipokine levels differ significantly compared to control groups of women matched for BMI without the condition (85).

The available data are limited and very often the research results are mutually exclusive (**Table 1**). In the literature on the subject, we find mainly studies on women of reproductive age. Moreover, another fundamental limitation of studies on the influence of adipokines on the etiopathogenesis of PCOS in girls is the small size of the studied populations, as well as the heterogeneous inclusion criteria for the study, which makes it difficult to interpret and compare the obtained results with the data available in the literature. Further investigation in this direction is needed, because considering the increasing incidence of overweight and obesity in modern society, it is all the more important to highlight this problem and a full understanding of the pathophysiology of PCOS will allow us to identify new effective therapeutic targets for this endocrinopathy from an early age.

**Table 1:** Serum levels of adipokines in adolescents with PCOS.

Adipokine	The concentration of adipokines in the blood of adolescents with PCOS	References
Leptin	Similar levels in the group of girls with obesity and PCOS compared to the control group with obesity alone Significantly higher levels in a group of adolescents with obesity and PCOS compared to normal-weight adolescents with PCOS	(17) (18)
Adiponectin	Lower levels in a group of adolescent girls with obesity and PCOS compared to a group of normal-weight girls with PCOS Lower levels among adolescent girls with PCOS compared to controls, but is independent of BMI	(32) (33)
Omentin	Lower omentin-1 levels in adolescent girls with obesity and PCOS compared to girls with obesity but without PCOS	(41)
Vaspin	Insignificantly elevated compared to BMI-matched controls	(12)
Resistin	Similar levels compared to BMI-matched controls	(36, 63)
Apelin	Similar levels compared to adolescents without PCOS	(80)
Visfatin	Similar levels compared to adolescents without PCOS	(80)

## References

[1] Pestel J Blangero F Watson J Pirola L Eljaafari A. Adipokines in obesity and metabolic-related-diseases. Biochimie. (2023) 212:48–59. doi: 10.1016/j.biochi.2023.04.008 37068579

[2] Kershaw EE Flier JS. Adipose tissue as an endocrine organ. J Clin Endocrinol Metab. (2004) 89:4005–20. doi: 10.1210/jc.2004-0305 15181022

[3] Fielder S Nickkho-Amiry M Seif MW. Obesity and menstrual disorders. Best Pract Res Clin Obstet Gynaecol. (2023) 89:102343. doi: 10.1016/j.bpobgyn.2023.102343 37279629

[4] Seif MW Diamond K Nickkho-Amiry M. Obesity and menstrual disorders. Best Pract Res Clin Obstet Gynaecol. (2015) 29:516–27. doi: 10.1016/j.bpobgyn.2014.10.010 25467426

[5] Ibáñez L de Zegher F. Polycystic ovary syndrome in adolescent girls. Pediatr Obes. (2020) 15:12586. doi: 10.1111/ijpo.12586 31663293

[6] Tayebi N Yazdanpanahi Z Yektatalab S Pourahmad S Akbarzadeh M. The relationship between body mass index (BMI) and menstrual disorders at different ages of menarche and sex hormones. J Natl Med Assoc. (2018) 110:440–7. doi: 10.1016/j.jnma.2017.10.007 30129516

[7] Joham AE Norman RJ Stener-Victorin E Legro RS Franks S Moran LJ. Polycystic ovary syndrome. Lancet Diabetes Endocrinol. (2022) 10:668–80. doi: 10.1016/S2213-8587(22)00163-2 35934017

[8] Ibáñez L de Zegher F. Adolescent PCOS: a postpubertal central obesity syndrome. Trends Mol Med. (2023) 29:354–63. doi: 10.1016/j.molmed.2023.02.006 36964058

[9] Anderson AD Solorzano CM McCartney CR. Childhood obesity and its impact on the development of adolescent PCOS. Semin Reprod Med. (2014) 32:202–13. doi: 10.1055/s-0034-1371092 PMC4103796 24715515

[10] Deligeoroglou E Tsimaris P. Menstrual disturbances in puberty. Best Pract Res Clin Obstet Gynaecol. (2010) 24:157–71. doi: 10.1016/j.bpobgyn.2009.11.001 20034856

[11] Prokopowicz Z Stupar S Zachurzok A Małecka-Tendera E. Sieć adipokin u dziewcząt z zespołem policystycznych jajników. Endokrynol Ped. (2013) 4:45–54.

[12] Cekmez F Cekmez Y Pirgon O Canpolat FE Aydinöz S Ipcioglu OM. Evaluation of new adipocytokines and insulin resistance in adolescents with polycystic ovary syndrome. Eur Cytokine Netw. (2011) 22:32–7. doi: 10.1684/ecn.2011.0279 21411410

[13] Kale-Gurbuz T Akhan SE Bastu E Telci A Iyibozkurt AC Topuz S. Adiponectin, leptin and ghrelin levels in obese adolescent girls with polycystic ovary syndrome. J Pediatr Adolesc Gynecol. (2013) 26:27–30. doi: 10.1016/j.jpag.2012.09.002 23158749

[14] Rajala MW Scherer PE. Minireview: The adipocyte at the crossroads of energy homeostasis, inflammation, and atherosclerosis. Endocrinology. (2003) 144:3765–73. doi: 10.1210/en.2003-0580 12933646

[15] Licinio J Caglayan S Ozata M O Yildiz B de Miranda PB O’Kirwan F. Phenotypic effects of leptin replacement on morbid obesity, diabetes mellitus, hypogonadism, and behavior in leptin-deficient adults. Proc Natl Acad Sci U.S.A. (2004) 101:4531–6. doi: 10.1073/pnas.0308767101 PMC384781 15070752

[16] Hausman GJ Barb CR Lents CA. Leptin and reproductive function. Biochimie. (2012) 94:2075–81. doi: 10.1016/j.biochi.2012.02.022 22980196

[17] Kedikova SE Sirakov MM Boyadzhieva MV. Leptin levels and adipose tissue percentage in adolescents with polycystic ovary syndrome. Gynecol Endocrinol. (2013) 29:384–7. doi: 10.3109/09513590.2012.752455 23350621

[18] Li L Feng Q Ye M He Y Yao A Shi K. Metabolic effect of obesity on polycystic ovary syndrome in adolescents: a meta-analysis. J Obstet Gynaecol. (2017) 37:1036–47. doi: 10.1080/01443615.2017.1318840 28657375

[19] Y Al-Taee SM Al-Allaff RGM E Alnajafy L. The effect of leptin on the regulation of immune responses in women with polycystic ovary syndrome. Pak J Biol Sci. (2022) 25:715–24. doi: 10.3923/pjbs.2022.715.724 36098197

[20] Zukauskaite S Seibokaite A Lasas L Lasiene D Urbonaite B Kiesylyte J. Serum hormone levels and anthropometric characteristics in girls with hyperandrogenism. Medicina (Kaunas). (2005) 41:305–12. 15864003

[21] Kadowaki T Yamauchi T. Adiponectin and adiponectin receptors. Endocr Rev. (2005) 26:439–51. doi: 10.1210/er.2005-0005 15897298

[22] Weyer C Funahashi T Tanaka S Hotta K Matsuzawa Y Pratley RE. Hypoadiponectinemia in obesity and type 2 diabetes: close association with insulin resistance and hyperinsulinemia. J Clin Endocrinol Metab. (2001) 86:1930–5. doi: 10.1210/jcem.86.5.7463 11344187

[23] Haluzik M Parizkova J Haluzik MM. Adiponectin and its role in the obesity- -induced insulin resistance and related complications. Physiol Res. (2004) 53:123–9. 15046547

[24] Yamamoto Y Hirose H Saito I Tomita M Taniyama M Matsubara K. Correlation of the adipocyte derived protein adiponectin with insulin resistance index, serum high-density lipoproteincholesterol in the Japanese population. Clin Sci. (2002) 103:137–42. doi: 10.1042/cs1030137 12149104

[25] Huang KC Chen CL Chuang LM Ho SR Tai TY Yang WS. Plasma adiponectin levels and blood pressures in nondiabetic adolescent females. J Clin Endocrinol Metab. (2003) 88:4130–4. doi: 10.1210/jc.2003-030158 12970275

[26] Maliqueo M Galgani JE Pérez-Bravo F Echiburú B Ladrón de Guevara A Crisosto N. Relationship of serum adipocyte-derived proteins with insulin sensitivity and reproductive features in pre-pubertal and pubertal daughters of polycystic ovary syndrome women. Eur J Obstet Gynecol Reprod Biol. (2012) 161:56–61. doi: 10.1016/j.ejogrb.2011.12.012 22277163

[27] Spranger J Möhlig M Wegewitz U Ristow M Pfeiffer AF Schill T. Adiponectin is independently associated with insulin sensitivity in women with polycystic ovary syndrome. Clin Endocrinol. (2004) 61:738–46. doi: 10.1111/j.1365-2265.2004.02159.x 15579189

[28] Comim VF Hardy K Franks S. Adiponectin and its receptors in the ovary: further evidence for a link between obesity and hyperandrogenism in polycystic ovary syndrome. PloS One. (2013) 8:80416. doi: 10.1371/journal.pone.0080416 PMC3832407 24260388

[29] Riestra P Garcia-Anguita A Ortega L Garcés C. Relationship of adiponectin with sex hormone levels in adolescents. Horm Res Paediatr. (2013) 79:83–7. doi: 10.1159/000346898 23429067

[30] Psilopanagioti A Papadaki H Kranioti EF Alexandrides TK Varakis JN. Expression of adiponectin and adiponectin receptors in human pituitary gland and brain. Neuroendocrinology. (2009) 89:38–47. doi: 10.1159/000151396 18698133

[31] Cankaya S Demir B Aksakal SE Dilbaz B Demirtas C Goktolga U. Insulin resistance and its relationship with high molecular weight adiponectin in adolescents with polycystic ovary syndrome and a maternal history of polycystic ovary syndrome. Fertil Steril. (2014) 102:826–30. doi: 10.1016/j.fertnstert.2014.05.032 24973036

[32] Pinhas-Hamiel O Singer S Pilpel N Koren I Boyko V Hemi R. Adiponectin levels in adolescent girls with polycystic ovary syndrome (PCOS). Clin Endocrinol (Oxf). (2009) 71:823–7. doi: 10.1111/j.1365-2265.2009.03604.x 19389110

[33] Yasar L Ekin M Gedikbasi A Erturk AD Savan K Ozdemir A. Serum adiponectin levels in high school girls with polycystic ovary syndrome and hyperandrogenism. J Pediatr Adolesc Gynecol. (2011) 24:90–3. doi: 10.1016/j.jpag.2010.11.003 21190873

[34] Toulis KA Goulis DG Farmakiotis D Georgopoulos NA Katsikis I Tarlatzis BC. Adiponectin levels in women with polycystic ovary syndrome: a systematic review and a meta-analysis. Hum Reprod Update. (2009) 15:297–307. doi: 10.1093/humupd/dmp006 19261627

[35] Yilmaz M Bukan N Demirci H Oztürk C Kan E Ayvaz G. Serum resistin and adiponectin levels in women with polycystic ovary syndrome. Gynecol Endocrinol. (2009) 25:246–52. doi: 10.1080/09513590802653833 19408174

[36] Güven A Ozgen T Aliyazicioğlu Y. Adiponectin and resistin concentrations after glucose load in adolescents with polycystic ovary syndrome. Gynecol Endocrinol. (2010) 26:30–8. doi: 10.3109/09513590903159540 19639497

[37] Mirza SS Shafique K Shaikh AR Khan NA Qureshi MA. Association between circulating adiponectin levels and polycystic ovarian syndrome. J Ovarian Res. (2014) 7:18. doi: 10.1186/1757-2215-7-18 24502610 PMC3928320

[38] Radzik-Zając J Wytrychowski K Wiśniewski A Barg W. The role of the novel adipokines vaspin and omentin in chronic inflammatory diseases. Pediatr Endocrinol Diabetes Metab. (2023) 29:48–52. doi: 10.5114/pedm.2022.121371 36734393 PMC10226453

[39] Yang RZ Lee MJ Hu H Pray J Wu HB Hansen BC. Identification of omentin as a novel depot-specific adipokine in human adipose tissue: Possible role in modulating insulin action. Am J Physiol Endocrinol Metab. (2006) 290:1253–61. doi: 10.1152/ajpendo.00572.2004 16531507

[40] Catli G Anik A Abaci A Kume T Bober E. Low omentin-1 levels are related with clinical and metabolic parameters in obese children. Exp Clin Endocrinol Diabetes. (2013) 121:595–600. doi: 10.1055/s-0033-1355338 24085389

[41] Özgen IT Oruçlu S Selek S Kutlu E Guzel G Cesur Y. Omentin-1 level in adolescents with polycystic ovarian syndrome. Pediatr Int. (2019) 61:147–51. doi: 10.1111/ped.13761 30566253

[42] Choi JH Rhee EJ Kim KH Woo HY Lee WY Sung KC. Plasma omentin-1 levels are reduced in non-obese women with normal glucose tolerance and polycystic ovary syndrome. Eur J Endocrinol. (2011) 165:789–96. doi: 10.1530/EJE-11-0375 21865408

[43] Orlik B Madej P Owczarek A Skalba P Chudek J Olszanecka-Glinianowicz M. Plasma omentin and adiponectin levels as markers of adipose tissue dysfunction in normal weight and obese women with polycystic ovary syndrome. Clin Endocrinol (Oxf). (2014) 81:529–35. doi: 10.1111/cen.12381 24392647

[44] Tan BK Adya R Farhatullah S Lewandowski KC O’Hare P Lehnert H. Omentin-1, a novel adipokine, is decreased in overweight insulin-resistant women with polycystic ovary syndrome: Ex vivo and *in vivo* regulation of omentin-1 by insulin and glucose. Diabetes. (2008) 57:801–8. doi: 10.2337/db07-0990 18174521

[45] Kurowska P Mlyczyńska E Dawid M Jurek M Klimczyk D Dupont J. Review: vaspin (SERPINA12) expression and function in endocrine cells 2021. Cells. (2021) 10:1710. doi: 10.3390/cells10071710 34359881 PMC8307435

[46] Gajewska J Kuryłowicz A Mierzejewska E Ambroszkiewicz J Magdalena Chełchowska M Weker H. Are omentin rs2274907 and vaspin rs2236242 gene polymorphisms related to body composition, lipid profile and other adipokines in prepubertal healthy children? Endocr Res. (2020) 45:24–31. doi: 10.1080/07435800.2019.1630842 31204527

[47] Özkan EA Sadigov A Öztürk O. Evaluation of serum omentin-1, vaspin, leptin, adiponectin levels in obese/overweight children and their relationship with non-alcoholic fatty liver disease. Clin Nutr Res. (2022) 11:194–203. doi: 10.7762/cnr.2022.11.3.194 35949560 PMC9348910

[48] Reinehr T Roth CL. Inflammation markers in type 2 diabetes and the metabolic syndrome in the pediatric population. Curr Diabetes Rep. (2018) 18:131. doi: 10.1007/s11892-018-1110-5 30338401

[49] Buyukinan M Atar M Can U Pirgon O Guzelant A Deniz I. The association between serum vaspin and omentin-1 levels in obese children with metabolic syndrome Muammer Buyukinan. Metab Syndr Relat Disord. (2018) 16:76–81. doi: 10.1089/met.2017.0133 29319392

[50] Salama HM Galal A Motawie AA Kamel AF Ibrahim DM Aly AA. Adipokines vaspin and visfatin in obese children. Open Access Maced J Med Sci. (2015) 3:563–6. doi: 10.3889/oamjms.2015.123 PMC4877888 27275288

[51] Zlatkina V. Vaspin levels and carbohydrate status in young patients with hypertension and obesity. Georgian Med News. (2016) 259:18–22. 27845280

[52] Ko BJ Lee M Park HS Han K Cho GJ Hwang TG. Elevated vaspin and leptin levels are associated with obesity in prepubertal Korean children. Endocr J. (2013) 60:609–16. doi: 10.1507/endocrj.ej12-0384 23318644

[53] Suleymanoglu S Tascilar E Pirgon O Tapan S Meral C Abaci A. Vaspin and its correlation with insulin sensitivity indices in obese children. Diabetes Res Clin Pract. (2009) 84:325–8. doi: 10.1016/j.diabres.2009.03.008 19356820

[54] Körner A Neef M Friebe D Erbs S Kratzsch J Dittrich K. Vaspin is related to gender, puberty and deteriorating insulin sensitivity in children. Int J Obes (Lond). (2011) 35:578–86. doi: 10.1038/ijo.2010.196 20856257

[55] Martos-Moreno GÁ Kratzsch J Körner A Barrios V Hawkins F Kiess W. Serum visfatin and vaspin levels in prepubertal children: effect of obesity and weight loss after behavior modifications on their secretion and relationship with glucose metabolism. Int J Obes (Lond). (2011) 35:1355–62. doi: 10.1038/ijo.2010.280 21266955

[56] Lee MK Jekal Y Jee-Aee Im AA Kim E Lee SH Park JH. Reduced serum vaspin concentrations in obese children following short-term intensive lifestyle modification. Clin Chim Acta. (2010) 411:381–5. doi: 10.1016/j.cca.2009.12.003 20018186

[57] Park HK Kwak MK Kim HJ Ahima RS. Linking resistin, inflammation, and cardiometabolic diseases. Korean J Intern Med. (2017) 32:239–47. doi: 10.3904/kjim.2016.229 PMC5339472 28192887

[58] Ntaios G Gatselis NK Makaritsis K Dalekos GN. Adipokines as mediators of endothelial function and atherosclerosis. Atherosclerosis. (2013) 227:216–21. doi: 10.1016/j.atherosclerosis.2012.12.029 23332774

[59] Sartori C Lazzeroni P Merli S Patianna VD Viaroli F Cirillo F. From placenta to polycystic ovarian syndrome: the role of adipokines. Mediators Inflamm. (2016) 2016:4981916. doi: 10.1155/2016/4981916 27746590 PMC5056282

[60] Raeisi T Rezaie H Darand M Taheri A Garousi N Razi B. Circulating resistin and follistatin levels in obese and non-obese women with polycystic ovary syndrome: A systematic review and meta-analysis. PLoS One. (2021) 16:e0246200. doi: 10.1371/journal.pone.0246200 33740002 PMC7978365

[61] Khademi Z Pourreza S Hamedi-Shahraki S Amirkhizi F. Association between selenium and circulating adipokine levels in patients with polycystic ovary syndrome. Biol Trace Elem Res. (2024) 202:3442–8. doi: 10.1007/s12011-023-03935-2 37910262

[62] Mehrabani S Arab A Karimi E Nouri M Mansourian M. Blood circulating levels of adipokines in polycystic ovary syndrome patients: A systematic review and meta-analysis. Reprod Sci. (2021) 28:3032–50. doi: 10.1007/s43032-021-00709-w 34472034

[63] Bideci A Camurdan MO Yeşilkaya E Demirel F Cinaz P. Serum ghrelin, leptin and resistin levels in adolescent girls with polycystic ovary syndrome. J Obstet Gynaecol Res. (2008) 34:578–84. doi: 10.1111/j.1447-0756.2008.00819.x 18937712

[64] Kleinz MJ Skepper JN Davenport AP. Immunocytochemical localisation of the apelin receptor, APJ, to human cardiomyocytes, vascular smooth muscle and endothelial cells. Regul Pept. (2005) 126:233–40. doi: 10.1016/j.regpep.2004.10.019 15664671

[65] Lee DK Cheng R Nguyen T Fan T Kariyawasam AP Liu Y. Characterization of apelin, the ligand for the APJ receptor. J Neurochem. (2000) 74:34–41. doi: 10.1046/j.1471-4159.2000.0740034.x 10617103

[66] Kotwica T Kosmala W. Rola apeliny w fizjologii i chorobach układu sercowo- naczyniowego. Pol Przegl Kardiol. (2008) 10:55–8.

[67] Strażyńska A Bryl W Hoffman K Pupek-Musialik D. Apelina w patogenezie chorób sercowo-naczyniowych — aktualny stan wiedzy. Nadciśn Tęt. (2009) 13:417–21.

[68] De Mota N Reaux-Le Goazigo A El Messari S Chartrel N Roesch D Dujardin C. Apelin, a potent diuretic neuropeptide counteracting vasopressin actions through inhibition of vasopressin neuron activity and vasopressin release. Proc Natl Acad Sci U S A. (2004) 101:10464–9. doi: 10.1073/pnas.0403518101 PMC478592 15231996

[69] Dray C Knauf C Daviaud D Waget A Boucher J Buléon M. Apelin stimulates glucose utilization in normal and obese insulin-resistant mice. Cell Metabol. (2008) 8:437–45. doi: 10.1016/j.cmet.2008.10.003 19046574

[70] Kolahdouzi S Baghadam M Kani-Golzar FA Saeidi A Jabbour G Ayadi A. Progressive circuit resistance training improves inflammatory biomarkers and insulin resistance in obese men. Physiol Behav. (2019) 205:15–21. doi: 10.1016/j.physbeh.2018.11.033 30503849

[71] Hu L Deeney JT Nolan CJ Peyot ML Ao A Richard AM. Regulation of lipolytic activity by longchain acyl-coenzyme A in islets and adipocytes. Am J Physiol Endocrinol Metab. (2005) 289:1085–92. doi: 10.1152/ajpendo.00210.2005 16091387

[72] Ziora K Oswiecimska J Swietochowska E Ziora D Ostrowska Z Stpjewska M. Assessment of serum apelin levels in girls with anorexia nervosa. J Clin Endocrinol Metab. (2010) 95:2935–41. doi: 10.1210/jc.2009-1958 20382684

[73] Daviaud D Boucher J Gesta S Dray C Guigne C Quilliot D. TNF-a up-regulates apelin expression in human and mouse adipose tissue. FASEB J. (2006) 20:1528–30. doi: 10.1096/fj.05-5243fje 16723381

[74] Sorli SC Le Gonidec S Knibiehler B Audigier Y. Apelin is a potent activator of tumour neoangiogenesis. Oncogene. (2007) 26:7692–9. doi: 10.1038/sj.onc.1210573 17563744

[75] Zheng XD Huang Y Li H. Regulatory role of apelin-13-mediated PI3K/AKT signaling pathway in the glucose and lipid metabolism of mouse with gestational diabetes mellitus. Immunobiology. (2021) 226:152135. doi: 10.1016/j.imbio.2021.152135 34521048

[76] Choi YS Yang HI Cho S Jung JA Jeon YE KIM HY. Serum asymmetric dimethylarginine, apelin, and tumor necrosis factor-α levels in nonobese women with polycystic ovary syndrome. Steroids. (2012) 77:1352–8. doi: 10.1016/j.steroids.2012.08.005 22944040

[77] Chang C-Y Tsai Y-C Lee C-H Chan T-F Wang S-H Su J-H. Lower serum apelin levels in women with polycystic ovary syndrome. Fertil Steril. (2011) 95:2520–3. doi: 10.1016/j.fertnstert.2011.04.044 21575945

[78] Altinkaya SÖ Nergiz S Küçük M Yüksel H. Apelin levels in relation with hormonal and metabolic profile in patients with polycystic ovary syndrome. Eur J Obstet Gynecol Reprod Biol. (2014) 176:168–72. doi: 10.1016/j.ejogrb.2014.02.022 24642195

[79] Olszanecka-Glinianowicz M Madej P Nylec M Owczarek A Szanecki W Skałba P. Circulating apelin level in relation to nutritional status in polycystic ovary syndrome and its association with metabolic and hormonal disturbances. Clin Endocrinol (Oxf). (2013) 79:238–42. doi: 10.1111/cen.12120 23199261

[80] Ruan X Li M Min M Ju R Wang H Xu Z. Plasma visfatin and apelin levels in adolescents with polycystic ovary syndrome. Gynecol Endocrinol. (2023) 39:2216807. doi: 10.1080/09513590.2023.2216807 37248950

[81] Benk Silfeler D Gokce C Keskin Kurt R Yilmaz Atilgan N Ozturk OH Turhan E. Does polycystic ovary syndrome itself have additional effect on apelin levels? Obstet Gynecol Int. (2014) 2014:536896. doi: 10.1155/2014/536896 25374607 PMC4206935

[82] Hosoya M Kawamata Y Fukusumi S Fujii R Habata Y Hinuma S. Molecular and functional characteristics of APJ. Tissue distribution of mRNA and interaction with the endogenous ligand apelin. J Biol Chem. (2000) 275:21061–7. doi: 10.1074/jbc.M908417199 10777510

[83] Gören K Sağsöz N Noyan V Yücel A Cağlayan O Bostancı MSühha. Plasma apelin levels in patients with polycystic ovary syndrome. J Turk Ger Gynecol Assoc. (2012) 13:27–31. doi: 10.5152/jtgga.2011.74 24627671 PMC3940220

[84] Hasan MM Abd El Hameed AA. Serum adipokine (apelin) in lean and obese polycystic ovary syndrome patients before and after metformin treatment. Middle East Fertility Soc J. (2018) 23:315–8. doi: 10.1016/j.mefs.2018.04.003

[85] Bril F Ezeh U Amiri M Hatoum S Pace L Chen YH. Adipose tissue dysfunction in polycystic ovary syndrom. J Clin Endocrinol Metab. (2023) 109:10–24. doi: 10.1210/clinem/dgad356 37329216 PMC10735305

